# Experimental Study on Rats with Critical-Size Bone Defects Comparing Effects of Autologous Bone Graft, Equine Bone Substitute Bio-Gen^®^ Alone or in Association with Platelet-Rich Fibrin (PRF)

**DOI:** 10.3390/polym16111502

**Published:** 2024-05-25

**Authors:** Petru Ciobanu, Mihai Danciu, Andrei Pascu, Ioannis Gardikiotis, Norin Forna, Mihnea Theodor Sirbu, Anca-Elena Calistru, Bogdan Puha, Bogdan Veliceasa, Paul-Dan Sirbu

**Affiliations:** 1 Department of Surgery II—Orthopedics and Traumatology, Grigore T. Popa University of Medicine and Pharmacy of Iasi, 700454 Iasi, Romania; 2 Department of Morphofunctional Sciences I—Morphopathology, Grigore T. Popa University of Medicine and Pharmacy of Iasi, 700454 Iasi, Romania; 3 Advanced Center for Research and Development in Experimental Medicine, Grigore T. Popa University of Medicine and Pharmacy of Iasi, 700454 Iasi, Romania; 4 Faculty of Medicine, Grigore T. Popa University of Medicine and Pharmacy of Iasi, 700454 Iasi, Romania; 5 Research Institute for Agriculture and Environment, Iasi University of Life Science, 700490 Iasi, Romania

**Keywords:** autologous bone graft, critical-size bone defect, platelet-rich plasma, Bio-Gen^®^, µCT, histomorphometry

## Abstract

Background: A critical-sized bone defect (CsBD) is considered one that will not heal spontaneously and requires reconstruction. This study aims to compare the results of using different bone reconstructive techniques and to study the potential of platelet-rich fibrin (PRF) to enhance the healing properties of a bone substitute (BS). Methods: In this experimental study on rats, the treatment of critical-sized bone defects was carried out by analysing four groups: a control group in which the bone defect was left empty; a group treated with Bio-Gen^®^; another group in which the defect was treated with PRF in combination with Bio-Gen^®^; and the last that was treated with autologous bone graft (ABG). The defects were evaluated by microcomputed tomography (µCT) and then histomorphometrically. Results: From both the histological and imagistic point of view, the best results were registered in the ABG group, followed by the group treated with Bio-Gen^®^ with PRF, Bio-Gen^®^ group, and control group, with statistically significant differences. Conclusions: A 5 mm defect in the rat radius can be considered critical. ABG showed the best results in treating the bone defect. PRF significantly enhanced the efficacy of Bio-Gen^®^.

## 1. Introduction

A critical-sized bone defect (CsBD) is considered one that will not heal spontaneously. CsBDs require reconstruction, and the current “gold standard” is still autologous bone grafting. Due to the disadvantages, risks, and costs involved in autologous graft harvesting and transfer, in the last decades, multiple alternative techniques have been extensively studied and put into practice [1,2].

Following the bone demineralization processes published for the first time by Urist, the matrix is rich in type I collagen and the non-collagenous proteins BMP, TGF-β, IGF, and FGF remain at the cortical bone level. However, the remaining matrix lacks mechanical support, having main osteoconductive and osteoinductive roles and less fixation in the initial post-procedural phases. The osteoinductive capacity of the bone substitute obtained is dependent on the levels of BMP2 and BMP7 found in its composition as the main growth factors. Although the allograft undergoes the same technological process, osteoconductivity is different due to differences in bone quality and in human factors involved in the harvesting, processing, and storage course of the demineralized bone matrix [3,4].

Bioteck^®^ has developed the exclusive deantigenation process named Zymo-Teck^®^ by the manufacturer. The Zymo-Teck^®^ process, unlike other methods based on high-temperature treatments or the use of chemical solvents to completely demineralize the bone, uses natural enzymes and proteins capable of the controlled and selective removal of antigens and proteins that can cause an immunological reaction, making the tissues completely biocompatible and free of treatment residues. Zymo-Teck^®^ also preserves useful molecules such as collagen in its natural structure and, operating at controlled temperatures, does not alter the structural characteristics of the tissues according to the manufacturer’s descriptions. After the complete deantigenization of the bone graft by enzymatic processes, the material is sterilized by β-ray irradiation. Bioteck^®^ bone substitutes are a completely preserved extracellular matrix and therefore undergo a complete remodelling process within the physiological time frame. The intact and unmodified collagen component allows the preservation of the strength characteristics of natural bone. Furthermore, in its native structure, bone collagen possesses all its distinctive effects, including acting as a co-activator of endogenous growth factors and as an underlying layer for osteoblast cell adhesion, consequently creating a favourable physiological environment for promoting bone regeneration through high osteoconductivity. The presence of bone collagen in Bioteck^®^ grafts is also visible under polarized light, and the collagen fibres, having a usual structure, display the characteristic refraction that makes them appear brighter.

Leukocyte-rich PRF or L-PRF was described for the first time in France in 2000, and is considered a new generation platelet concentrate that received special attention in the following years [5]. The PRF preparation technique does not require anticoagulant or activating agent, thus using pure blood for centrifugation. The preparation protocol is very simple: a 10 mL blood sample is collected without anticoagulant in a sterile tube, which is then immediately centrifuged at 3000 rpm for 10 min [6].

The success of this technique depends largely on the speed with which the blood is collected and put into the centrifuge, as the coagulation process of the sample will begin almost as soon as the blood comes into contact with the wall of the container. In conclusion, the PRF protocol makes it possible to obtain a fibrin clot with a high platelet load through a simple technique. Compared to PRP, PRF has several advantages:

- The preparation is simpler and more natural as it does not require the addition of an anticoagulant [7].
- Due to the three-dimensional structure of the fibrin clot, it may well protect growth factors from proteolysis.
- It causes a slower and prolonged release of growth factors compared to PRP.

The 3D structure of fibrin can be used as a scaffold for neoangiogenesis and has an osteoconductive role [8].

Although the published results regarding the efficiency of PRF or other concentrates of platelets (CPs) are variable, unpredictable, and debatable, their association with other synthetic or biological products improves the properties of CPs and the substitute, acting synergistically for bone regeneration. Multiple studies report favourable results of combining CPs with other bone substitutes, such as tricalcium phosphate (TCP), polycaprolactone, autograft, allograft, and bone matrix of xenologous origin [9,10,11,12,13,14].

Despite the advantages of the techniques mentioned above, the autologous bone graft remains the method of choice in the management of bone defects, being the only material that combines osteoconductive, osteoinductive and osteogenic capabilities. They are fully biocompatible products and safe from the immunology and disease transmission points of view, as they are harvested from the same individual; however, this method is associated with a number of disadvantages that cannot be neglected [15,16,17,18,19,20].

In this experimental study on rats, the treatment of critical bone defects was carried out by analysing four groups: a control group in which the bone defect was left empty; a group in which the defect was treated with Bio-Gen^®^, another group in which the defect was treated with PRF in combination with Bio-Gen^®^, and a group in which the defect was treated with autologous bone graft.

To our knowledge, this is the first study combining PRF with a deantigenated equine bone substitute such as Bio-Gen^®^ for CsBD treatment.

## 2. Materials and Methods

The experiment was conducted at the Advanced Center for Research and Development in Experimental Medicine (CEMEX) of Grigore T. Popa University of Medicine and Pharmacy of Iasi. The Ethics Committee of the University approved the study (Nr. 92/21.02.2021). The veterinary authority (DSVSA) approved the project plan on rats (Nr. 47/18.02.2022). The experiment was carried out in accordance with the requirements provided by Directive 2010/63 of the European Parliament regarding the protection of animals used for scientific purposes.

This experiment was carried out by forming four research groups, and to reduce the number of animals used, bone defects were made on both radial bones; thus, the left and right limbs were part of different experimental groups. Twenty-seven animals were used in this experiment to create two groups of twelve animals each; three animals were blood donors for the preparation of PRF. In the first group of 12 animals, the bone defect of the right thoracic limb was part of the group in which the defect was treated with bone substitute of equine origin, called the BS group (bone substitute); the right limb formed the group in which the bone defect was treated with autologous cancellous bone graft, called the ABG (autologous bone graft) group. In the second batch of 12 animals, the right limbs formed the group named BS + PRF, where the bone defect was treated with a mix of the same bone substitute and PRF, and the left limbs formed the blank group, in which the radial bone defects was left empty, serving as a control group.

PRF was prepared according to the protocol described by Choukroun et al. in 2000 [5]. Immediately after collection, fresh blood was rapidly transferred into a glass-walled tube specially designed for the preparation of PRF to be centrifuged without the use of additives with the purpose of anticoagulation or activation of the final product upon use. Centrifugation was performed according to the protocol described by Choukroun et al. in a single step at 710 g, 2400 rpm, for 8 min in a horizontal position for a minimum mechanical aggression of the cells, but also for a maximum separability. After centrifugation, the three layers were therefore obtained: the sediment formed by erythrocytes; the middle layer rich in platelets, leukocytes, vascular endothelial growth factor (VEGF), and other growth factors; and the supernatant formed by plasma poor in platelets and growth factors. Since coagulation of the centrifuged blood occurred simultaneously with centrifugation, the PRF was obtained in the form of a clot, and its separation from the other layers was performed by sectioning the clot with a scalpel. 

Centrifugation was performed using a Hettich Universal 32R with the parameters mentioned in the protocol. The middle layer, formed by the PRF clot, was combined with the Bio-Gen^®^ corticosponge granule mix in an approximate ratio of 1:1, and the composition obtained by manual mixing for homogenization was then introduced into the radius bone defect of Lot 3 (BS + PRF).

Before the induction of anaesthesia, the animals were weighed to administer an anaesthetic dose according to weight and to avoid the risk of overdose. The weight of the animals was between 201 g and 285 g, with an average of 242 g.

To minimize animal suffering, the induction of anaesthesia was performed with isoflurane (ISOFLUTEK^®^ Laboratorios Karizoo S.A., An Alivira Group Company, Barcelona, Spain). After the induction of anaesthesia, the area to be operated on was prepared by trimming it with an electric clipper. Fifteen minutes before the start of the surgery, a dose of antibiotic with amoxicillin/clavulanic acid (SYNULOX, Zoetis Belgium S.A., Louvain-la-neuve, Belgium) was administered (intramuscular injection) at 100 mg/kg (single dose of 1.7 mL for a 250 g animal) for prophylactic purposes.

Afterwards, to maintain general anaesthesia, a combination of 100 mg/mL ketamine (VetaKetam, SC Maravet SRL, Lublin, Poland) mixed with 2% xylazine (Xylazine BIO, Bioveta a.s., Ivanovice na Hané, Czech Republic) was administered intraperitoneally (ketamine/xylazine: 9:1) by administering 0.3 mL to a 250 g animal. 

**Figure 1:**
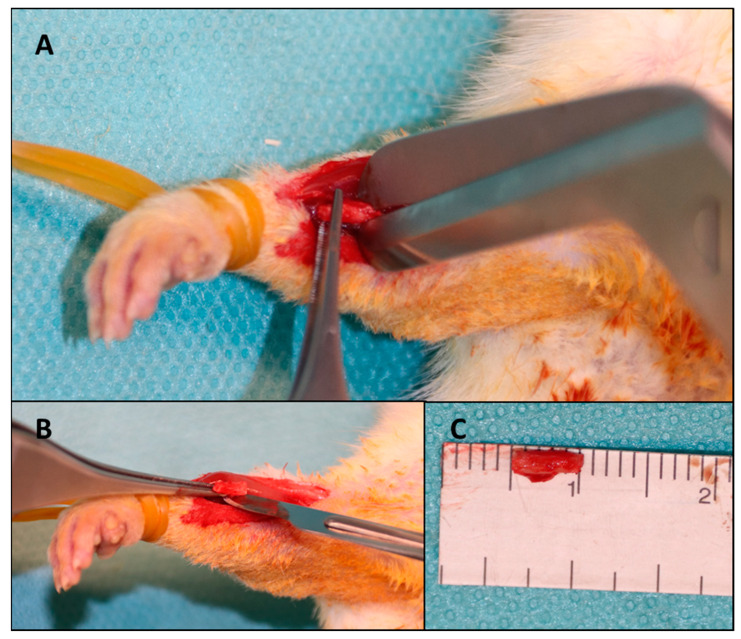
The surgical steps for making the CsBD. (**A**) bone sectioning. (**B**) interosseus membrane sectioning. (**C**) bone defect dimensions confirmation.

The skin was prepared with 10% Betadine^®^ (EGIS PHARMACEUTICALS PLC, Körmend, Hungary) with the rat fixed with both thoracic limbs on an operating table connected to a continuous monitoring and heating system to avoid hypothermia. A 2 cm skin incision was made on the projection of the radius with a scalpel in a sterile manner. The intermuscular plane was identified for fine dissection in an avascular plane using fine dissecting scissors. When the radial bone plane was identified, the dissection resumed with the scalpel, with which the periosteum, muscle insertions, interosseous membrane and soft parts adhering to the 5 mm of bone from the middle portion of the radius diaphysis were detached (Figure 1).

After completely freeing the soft tissues and obtaining good visibility of the portion to make the osteotomy, 5 mm of bone was measured, and the osteotomy points were marked. Using a guillotine osteotome, the cut was performed in a precise manner to avoid injury to adjacent structures and to avoid comminution. The interosseous membrane adjacent to the bone defect was preserved to avoid limb destabilization and migration of the fractured bone ends; the interosseous membrane preventing the extraction of the osteotomized bone fragment was sectioned. After the creation of the bone defects, the protocol was different according to the batch with which the respective defect was treated.

Batch 1: BS—the Bio-Gen^®^ corticosponge granule mix was rehydrated in a 5 mL saline solution for several minutes according to the manufacturer’s recommendations, and then an appropriate amount of the composition was placed into the bone defect, matching the defect.

Batch 2: ABG—the bone fragment excised from the right radius of the same animal was first mechanically processed by shredding it to pieces similar to the bone substitute used (0.25 mm—1 mm), and then placed in a similar manner as described for the previous batch.

Batch 3: BS + PRF—PRF was prepared according to the technique previously described, and then mixed with an equal amount of Bio-Gen^®^ bone substitute with the same grain size until it resulted in a homogeneous paste. This composition was then inserted into the radial bone defect in the corresponding batch in a manner similar to the other groups.

Batch 4: Blank—after creating the bone defect under the same conditions already described, the bone defect remained empty.

After completing the surgery for each batch, the same protocol was followed. After treating the defect corresponding to the batch to which it belongs, the periosteum was repaired with a continuous suture with 5/0 Nylon. After that, the muscle planes were reconstructed to avoid material migration. Skin repair was then performed with 4/0 Nylon and a bitter spray was administered locally to avoid early thread biting that could cause dehiscence or infection.

At the end of the surgery, a dose of Dex-ketoprofen (Tador, BERLIN-CHEMIE MENARINI) of 1.25 mg per animal and an intraperitoneal dose of 2 mL of saline were administered intraperitoneally for volume rebalancing and post-operative analgesia. Next, the general state of the animal, the behaviour upon waking up, and the resumption of feeding was monitored. All animals were monitored daily until the end of the study for the following parameters:

- clinical behaviour;
- support on the thoracic limbs;
- the ability to feed;
- pain on palpation;
- oedema, hyperaemia, and secretions at the postoperative wound.

The study period was 8 weeks; at the end of this period, animals were euthanized by administering a lethal dose of ketamine and xylazine and were monitored for the reappearance of vital functions for 1 h. After euthanasia, the thoracic limbs were harvested and the soft parts around the ulna–radius complex were excised, keeping the elbow and radiocarpal joints intact, in order to not cause changes in the bone defect. The samples were subsequently fixed in a 10% formalin solution to be examined by imaging and histopathology.

The µCT scans were performed at the Research Institute for Agriculture and the Environment in Iași. The equipment used was a µCT, SkyScan 1273, from Bruker (Belgium). The samples, which were preserved in 10% formalin until analysis, were removed from the vials, gently dried with a tissue, and then fixed on the scanning table of the equipment. The same scanning protocol was used for all samples at a source voltage of 55 kV, source current of 272 µA, and a pixel size of 28.7 µm. To obtain better image quality, the samples were scanned at a full 360-degree rotation with a rotation step of 0.3 degrees, and the random motion parameter was set with an amplitude (in number of detection lines) of 10, which can reduce ring artifacts in the reconstructed cross-sections. To reduce beam strengthening effects, which occur due to preferential absorption by the edges of the sample, a 0.5 mm Al filter was used.

For the reconstruction of cross-sectional images, the program package of Vox CT Software Version 3.3.0 r1412, Copyright© Bruker microCT^®^ was used.

The samples taken from the subjects were examined macroscopically, and the soft parts with no role in the diagnosis were excised so that the fragments could be fitted into the working boxes with a thickness of no more than 4–5 mm; then they were fixed in a solution of 10% formalin. After fixation, the tissue was subjected to the decalcification process to facilitate the sectioning of the bone tissue with the microtome and to obtain optimal histological sections for analysis. After decalcification, the pieces were trimmed to fit into boxes and prepared for the paraffin embedding process. The obtained paraffin block was used to obtain histological sections with a thickness of 4 micrometres with a microtome, and later stained with haematoxylin and eosin.

The slides were obtained with a sagittal section through the preparation for the most appropriate exposure of the bone defect area and the capture of the entire healing process in the histological field. The slides were evaluated with optical microscopy by analysing the following elements: the composition of the callus (bony, cartilaginous, or fibrous) and the percentage of the component tissues; angiogenesis; inflammatory infiltrate; giant cell foreign body reaction; the remodelling process; and the presence of foreign materials in the fracture focus. Variables were quantified by numerical data, which were reported as number of elements/1 mm^2^, and were statistically analysed.

The data were processed with IBM SPSS Statistics 21 software. To describe the batches by means of basic statistical indicators, the “Descriptive Statistics” section was used. To test the groups ABG, BS + PRF, BS and blank, for each of the post-treatment results, the non-parametric Mann–Whitney U and Kruskal–Wallis tests were applied. They are useful when the conditions of the t-test are not met in the given situation, not respecting the condition of normality.

## 3. Results

### 3.1. Histological Results

The histological examination aimed to qualitatively and quantitatively evaluate the newly formed callus in the bone defect in the four study groups. To distinguish healing quality in these four study groups, we have evaluated several parameters: the percentage of bone regeneration as a ratio between the volume of newly formed callus and the volume of the defect; the cellular composition of the callus by defining the percentages of fibrous, cartilage and bone cells; angiogenesis—by quantitating capillaries per mm^2^ in the callus; foreign body-type giant cell reaction—by counting macrophages; chronic inflammatory infiltrate—by counting lymphocytes; and bone remodelling—by counting osteoblasts and osteoclasts. The average values for each study group are displayed in Table 1.

The bone regeneration percentage is calculated as the ratio between the volume of the newly formed callus and the volume of the created 5 mm long bone defect.

Following the application of the Kruskal–Wallis test to the values obtained from the histological evaluation of the percentage of bone regeneration, the null hypothesis was rejected; significant differences were found between the values of the four study groups.

When applying the Mann–Whitney U test to the values obtained from the quantitative histological determination of bone regeneration, the study groups were compared pairwise. Statistically significant differences were noticed among the study groups and the results are displayed in descending order of difference in Table 2. In the tables showing the results of the application of the Mann–Whitney U test, the following abbreviations were used:

- Test value—represents the Wilcoxon W calculated value;
- Z statistic—represents the difference between the two groups;
- *p*-value—test significance.

Following the application of the Kruskal–Wallis test for the fibrous cell count identified by the histological examination, there was a 95% probability of observing significant differences among the four study groups.

When applying the Mann–Whitney U test to the results regarding the fibrous cell count in the callus, the study groups were compared pairwise. Statistically significant differences were noticed among the following groups: ABG and BS; ABG and control; ABG and BS + PRF; BS + PRF and BS; and BS + PRF and control. These results are displayed in descending order of difference in Table 3.

Following the application of the Kruskal–Wallis test for the cartilage cell count, there was a 95% probability of observing significant differences among the four study groups.

When applying the Mann–Whitney U test to the values obtained to determine the cartilage cell count in the callus, the study groups were compared pairwise. Statistically significant differences were noticed among the following study groups: ABG and BS; BS + PRF and BS; ABG and control; and BS + PRF and control. These results are displayed in descending order of difference in Table 4

Following the application of the Kruskal–Wallis test for the bone cell count, there was a 95% probability of observing significant differences among the four study groups.

When applying the Mann–Whitney U test to the values obtained to determine the bone cell count in the callus, the study groups were compared pairwise. Statistically significant differences were noticed among the following study groups: ABG and BS; ABG and control; ABG and BS + PRF; BS + PRF and BS; and BS + PRF and control. These results are displayed in descending order of difference in Table 5

Following the application of the Kruskal–Wallis test for angiogenesis, there was a 95% probability of observing significant differences among three study groups. It is important to note that the control group was excluded from the analysis of the angiogenesis parameter. 

When applying the Mann–Whitney U test to the values obtained to determine the blood vessel count in the callus, the study groups were compared pairwise. Statistically significant differences were observed among the following study groups: ABG and BS; ABG and BS+PRF; and BS + PRF and BS. These results are displayed in descending order of difference in Table 6.

Following the application of the Kruskal–Wallis test for the giant cell reaction, which was quantified as the number of inflammatory cells per mm^2^ determined by the histopathological examination, there was a 95% probability of observing significant differences among three study groups. It is important to note that the control group was excluded from the analysis of the variable giant cell reaction, as adjacent tissues migrated into the bone defect and it was difficult to determine an inflammatory reaction.

When applying the Mann–Whitney U test to the values obtained to determine the inflammatory cell count in the callus, the study groups were compared pairwise. Statistically significant differences were observed among the following study groups: ABG and BS; ABG and BS + PRF; and BS + PRF and BS. These results are displayed in descending order of difference in Table 7

Following the application of the Kruskal–Wallis test for the chronic inflammatory infiltrate, which was represented by the lymphocyte count per mm^2^, there was a 95% probability of observing significant differences among three study groups. It is important to note that the control group was excluded from the analysis of the inflammatory infiltrate variable characterized by lymphocytes due to the lack of a chronic inflammatory infiltrate.

Following the application of the Mann–Whitney U test for the chronic inflammatory infiltrate characterized by the presence of lymphocytes, the groups were compared pairwise and the values are displayed in Table 8. This table shows that there were significant differences for each comparison between groups, with the most important difference between the ABG and BS groups.

Following the application of the Kruskal–Wallis test to evaluate the bone remodelling process, as determined by osteoblast and osteoclast counts in a histological area of 1 mm^2^, there was a 95% probability of observing significant differences among the four study groups regarding the osteoblast and osteoclast counts present in the callus. 

Following the application of the Mann–Whitney U test for the bone remodelling process described by osteoblast and osteoclast counts, the groups were compared pairwise and the values are included in Table 9. We are hereby showing that the null hypothesis was not rejected only for the ABG and BS + PRF study groups; therefore, there was no significant difference between the two groups. At the same time, it is mentioned that the table is sorted the differences between the study groups in descending order, thus identifying the largest difference between the ABG and the control groups, and, in contrast, a minimal difference was identified between the ABG and BS + PRF groups, as displayed by the test significance.

**Figure 2:**
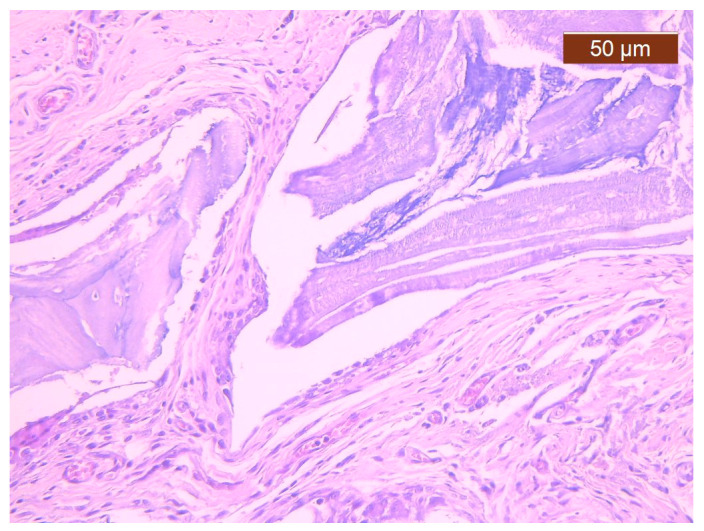
Fibrous callus displaying multiple capillaries, a giant cell reaction to Bio-Gen^®^ bone graft fragments, and rare lymphocytes. Histological snapshot from the BS group (magnification 20×). In the image, the giant cell reaction to the foreign body adjacent to a devitalized bone graft fragment and numerous capillary blood vessels can be observed.

In cases from the BS group, impaired healing was most commonly observed as the persistence of multiple devitalized bone fragments that induced a lymphoplasmacytic inflammatory reaction, which associated the giant cell reaction to the bone substitute used (Figure 2). At 8 weeks, the healing process was present as a fibrous callus that encompassed the bone fragments and joined the two free, post-fracture bone ends. Structurally, the callus consisted of collagen fibres that included fibrocytes that were more frequently identified compared to fibroblasts (which indicated a reduced metabolic activity and, consequently, delayed healing), often capillaries and lymphocytes. Overall, callus progression was related to a decrease in the vessel count within the initial fibro-vascular callus to facilitate the remodelling of cartilage and bone.

**Figure 3:**
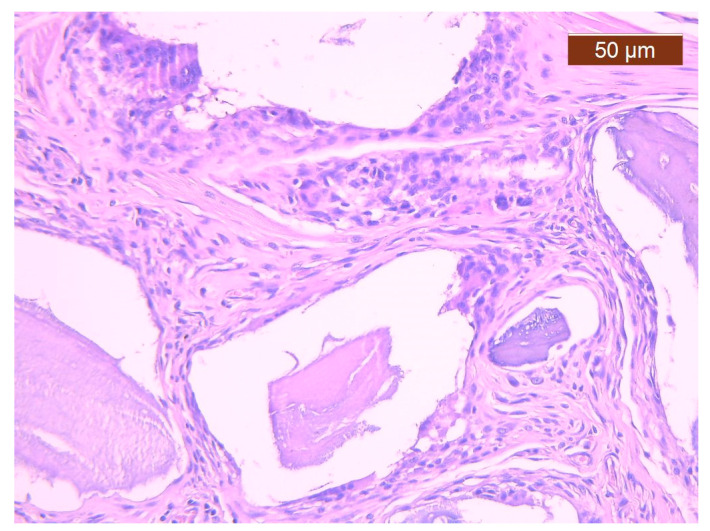
Fibrous callus that includes bone graft fragments, an inflammatory reaction and capillaries less frequently than in the BS group. Histological snapshot from the BS + PRF study group (magnification 20×).

For the BS + PRF group, a similar healing trend was observed, with the incorporation of devitalized bone fragments supported by fibrous connective tissue (Figure 3). As compared to BS cases, a reduced capillary density was identified (which may represent a closer trend towards healing). In contrast, the giant cell foreign body reaction was slightly more frequent for these cases compared to those treated with BS.

**Figure 4:**
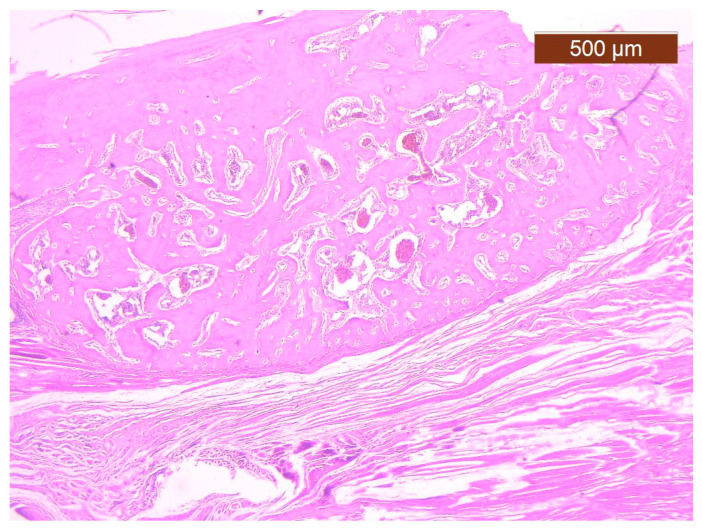
Trabecular bone callus from the ABG study group (magnification 2.5×).

The best healing output (from a histopathological perspective) was noticed for ABG cases, identifying aspects of callus progression. The fibrous component had significantly different aspects from the one observed in BS or BS + PRF cases, with a sharp lowering or lack of capillaries, lymphocytes, or foreign body reaction. A morphological transition from fibrous tissue was noticed with chondroplast formation following the synthesis and deposition of the cartilage matrix on collagen fibres (Figure 4). Subsequently, the cartilage matrix was filled with calcium salts to form the bone matrix, resulting in an immature bone that will be remodelled by tension forces.

### 3.2. Imaging Results

**Figure 5:**
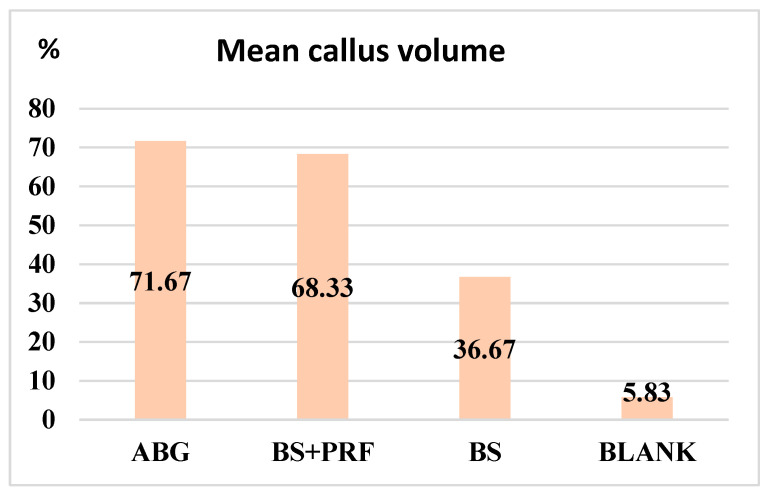
Representation of the mean callus volumes obtained from the μCT examination.

**Figure 6:**
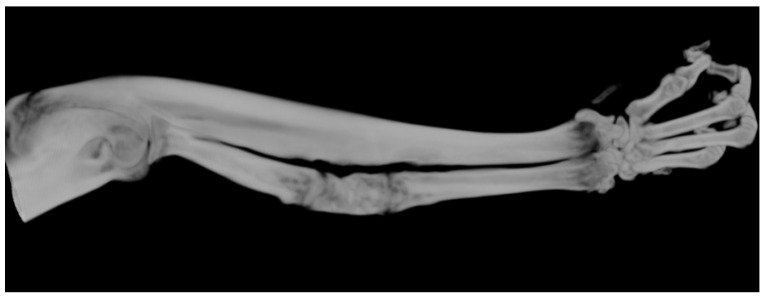
A 3D μCT image of the best healing sample from the ABG group, where the sample accounted for 100% bone healing; mineralization and radiotransparency similar to healthy bone may be observed.

**Figure 7:**
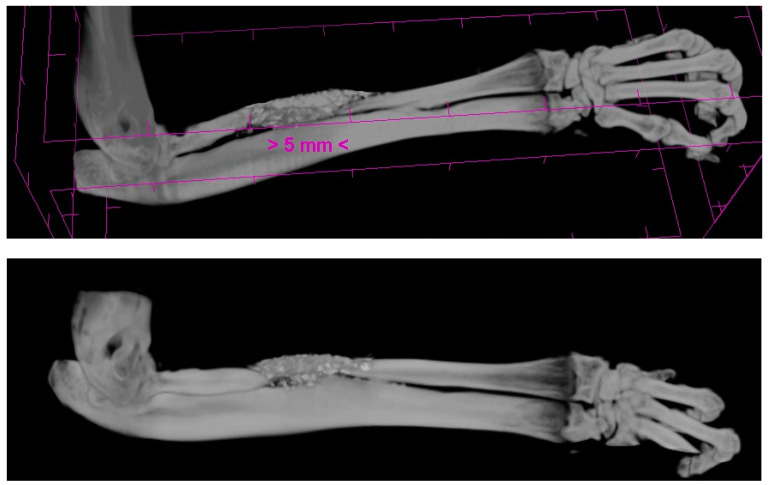
The 3D μCT images of samples from the BS + PRF group; both samples accounted for 90% bone healing.

**Figure 8:**
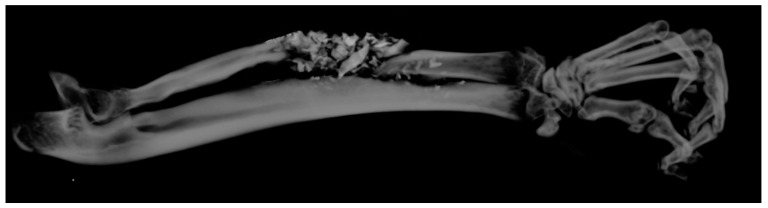
A 3D μCT image of a sample from the BS group with unstable healing due to sequestered bone replacement fragments, and the entire defect was unable to be healed. These images were assessed for about 30–40% of the bone defect, respectively.

**Figure 9:**
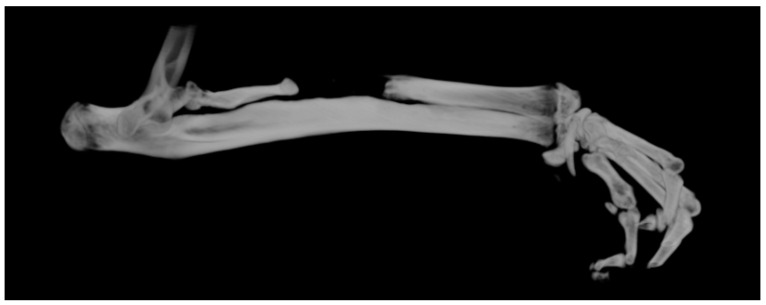
A 3D μCT image of a sample from the control group.

Following the imaging exploration by μCT and 3D image reconstruction through specialized software Vox CT Software Version 3.3.0 r1412, samples were randomly and blindly explored by two experienced medical imaging experts who generated a percentage score for the newly formed callus (Figure 5). Following averaging of the results from the two experts, percentage values for each sample were obtained, as displayed in Figure 4. These individual values were assigned as the total callus volume and were calculated as a percentage of the total volume of the induced bone defect. Selected images from each study group that were examined and evaluated after 3D reconstruction by Vox CT Software Version 3.3.0 r1412 are displayed in Figure 6, Figure 7, Figure 8 and Figure 9.

Following the application of the Kruskal–Wallis test for the calculated callus volume from the μCT examination, there was a 95% probability that there were significant differences among the four study groups, considering 48 observations.

Following the application of the Mann–Whitney U test to the total callus volume, the values shown in Table 10 were obtained by comparing the groups pairwise. This table shows that the null hypothesis was not rejected for the ABG and BS + PRF groups exclusively; therefore, there was no significant difference between the two study groups. Nevertheless, it is mentioned that the table is sorted descending order according to the differences between the study groups, thus the largest difference was identified between the BS and the control groups. Instead, the lowest differences were observed between the ABG and BS + PRF study groups.

## 4. Discussion

After going through scientific databases, we did not identify another study that tested the association between an equine corticocancellous bone substitute such as Bio-Gen^®^ and PRF in the treatment of CsBDs, as well as compared these results with autologous bone graft and an untreated defect of the same size; this was the first report in this field. The use of PRF in human surgical practice does not benefit from extensive evidence, but at least four studies can be identified in the literature, reporting good results when using PRF in bone defects in humans. A study of 11 people with mandibular bone defects due to drug-induced osteonecrosis shows excellent macroscopic results with rapid healing of the defects, better post-operative pain control and infection control using PRF membranes. All patients included in that study promoted good results at 12 to 36 months of follow-up [21].

Mauceri et al. reported a case of a critical bone defect resulting from the treatment of a maxillary cystic lesion that was successfully treated with PRF in combination with a bone substitute. The authors reported it as a feasible surgical technique in well-selected clinical situations of large bone defects [22].

Bone biomedical engineering techniques addressed in recent years substantially focus on combining bone substitutes with growth factors or osteoprogenitor cells. Wang et al. proved in an experimental study the effectiveness of combining bone marrow stem cell cultures with β-TCP in the treatment of segmental bone defects in goats, and these conclusions were drawn based on histological, imaging, and biomechanical tests [23]. The interest of researchers in the use of PRF independently or in combination with other osteoconductive products in the treatment of bone defects is high, hence the large number of publications, usually with preclinical studies. According to the results of a review of the literature by Idulhaq et al. in 2022, which aimed to identify all experimental studies in rats in which PRF was used independently or in combination with other bone healing promoters, PRF does not provide superior results to other products used, but the association of PRF with other bone substitutes guarantees clearly superior results [24]. In this study, the conclusion mentioned above is confirmed once again.

PRF is considered a second-generation platelet product used in the augmentation of bone regeneration processes but also in skin rejuvenation, treatment of alopecia, and management of complex wounds. Studies suggest that PRF alone may result in the healing of bone defects with a superior quality compared to cases without intervention, but show no advantage over demineralized bone matrix or allograft [25]. In the healing of bone defects, the synergistic effect of PRF was tested with other adjuvants such as polycaprolactone, TCP, bovine demineralized bone matrix (DBM), hydroxyapatite (HA), mesenchymal stem cells, adipocytes, GAO, or allograft [24,26]. In the present study, we combined PRF with a bone substitute of equine origin produced by enzymatic deantigenation and β-ray sterilization in the form of cortico-cancellous granule mix. This innovative bone substitute production technology shows promising results, and in the current study, it showed synergistic effects with PRF, significantly improving the ability to consolidate critical bone defects.

Histomorphometry is considered by many authors to be the technique of choice in assessing the quantity and quality of newly formed bone [27]. In the current study, autologous bone graft was shown to induce the most bone cell proliferation and promote bone remodelling by recruiting and multiplying osteoblasts and osteoclasts, while reducing inflammatory reactions and angiogenesis at 8 weeks. Additionally, in the ABG group, the largest amount of callus was recorded in the histological examination. Following the analysis of the same histological parameters, it was found that the BS group showed better results that were statistically significant compared to the blank group, and the BS + PRF group showed even better results, which were statistically supported, compared to the blank and BS groups.

When assessing the quality of bone healing, µCT plays an important role with high predictability. Parameters of interest in CT scans to assess bone healing are the bone mineral density and total callus volume. In the µCT imaging scans of the present study, we evaluated the consolidation of the bone defect by expressing the callus volume as a percentage of the volume of the segmental defect with a length of 5 mm. A review of the literature by Willems et al. identified five studies in which computed tomography was used as a method to assess the quality of bone healing and in which the correlation between the total callus volume and mechanical bioresistance was performed. Three of these showed a weak association between the callus volume and mechanical strength, and two studies reported a moderate association between the total callus volume and the results of mechanical tests, such as the three-point bend test or torsion test. Although the association between CT characteristics and mechanical bioresistance is a subject of debate in the literature, µCT examinations have better accuracy, use higher radiation doses, and have better spatial resolution compared to CT machines used in clinical practice [28,29,30,31,32,33,34].

Some authors consider the µCT evaluation as the method of choice for measuring the quantity and quality of newly formed bone in mandibular bone defects in small animal experimental studies. Combining a µCT examination with 3D image reconstruction can provide detailed, high-resolution information about the quantitative morphology of bone structure but also the quality of bone mineral density [35,36,37]. Another advantage of this investigation is that it is non-destructive and has the potential to provide accurate and high-resolution details of both intraosseous and intrerosseous bone [38].

Microcomputed tomography is also a widely used investigation in preclinical practice to evaluate different bone matrices and bone substitutes, but also their evolution, resorption, and integration into a bone defect and their ability to induce new bone synthesis. Based on these principles, we chose to use µCT investigation to evaluate the ability of the equine bone substitute Bio-Gen^®^ to consolidate a bone defect, as well as the ability of PRF to improve the osteoprogenitor properties of this biomaterial. However, it should be noted that this imaging investigation has limitations, among which are the inability to determine callus cellularity, bone tissue proliferation and vascularization, the bone remodelling capacity, and mineral accumulation rate [39].

## 5. Conclusions

The histopathological examination was focused on counting the cellularity relevant to bone healing and the body’s reaction to consolidate a bone defect. Therefore, the histological percentage of the callus relative to the defect, the number of bone cells, the number of cartilaginous cells, the number of fibrous cells, the number of blood vessels/mm^2^, the giant cell reaction, the chronic inflammatory infiltrate, and the remodelling process obtained through counting osteoblasts and osteoclasts were determined. Following the statistical analysis of these parameters, a series of conclusions were drawn:

- The measurement of the histological percentage of callus confirms the hypothesis that a segmental defect of 5 mm at the level of the radius of a rat can be considered critical, since it does not have the ability to heal spontaneously.
- The histological amount of the callus obtained was the highest in the ABG group, with statistically insignificant differences compared to the BS + PRF group and statistically significant differences compared to the BS and blank groups. In conclusion, the autologous bone graft determined the formation of the largest amount of callus, and PRF stimulated the Bio-Gen^®^ bone substitute in the quantitative production of callus, with statistically significant differences from the histological point of view.
- When determining the cellular composition of the callus by counting fibrous, cartilaginous and bone cells, the ABG group obtained the best values that were statistically significant compared to the other groups, with a predominance of bone cells, the most cartilaginous cells (compared to the other groups) and the least fibrous cells. The BS + PRF group was 2nd, but with a predominance of fibrosis, followed by cartilage and then bone cells. The BS + PRF group showed statistically significantly better values compared to the BS and blank groups, but at the same time, was statistically significant worse than the ABG group.
- Angiogenesis was quantified by counting callus blood vessels per mm^2^, and the results showed statistically significant differences between all groups, with the richest angiogenesis in the case of the BS group, followed by the BS + PRF group and then the ABG group.
- The giant cell reaction was the most intense in the BS group, followed by the BS + PRF group, but the difference between these two groups was not statistically significant. This hypothesis supports the fact that Bio-Gen^®^ induced a foreign body reaction, which was slightly attenuated by the application of PRF. In the ABG group, the giant cell reaction was reduced, with statistically significant differences compared to the other groups.
- The lymphocytic inflammatory infiltrate was the most abundant in the BS group, followed by the BS + PRF group and then the ABG group. The differences were statistically significant between all three groups involved in the comparison. This suggests that Bio-Gen^®^ can induce a chronic inflammatory reaction, which can be significantly attenuated by association with PRF.
- The bone remodelling process was evaluated by counting the osteoblasts and osteoclasts present in the callus. The remodelling process was shown to be the most effective in the BS + PRF group, followed by the ABG group, but without a statistically significant difference between them. The BS and blank groups presented the lowest values, with statistically significant differences compared to the other groups. The conclusion is that PRF can significantly influence the bone remodelling process by stimulating the activity of osteoblasts and osteoclasts.

From the imaging point of view, the best results for bone defect consolidation were obtained using the autologous bone graft, followed by the bone substitute in combination with PRF, the independent bone substitute, and the control. Non-significant statistical differences between the autologous bone graft and PRF bone substitute groups, suggest a similar quality of healing on the µCT examination. The bone substitute group showed poor healing with statistically significant differences compared with the first two groups. In the control group, healing was almost non-existent from an imaging point of view, which confirms the hypothesis of a critical bone defect that will not heal in the absence of reconstructive intervention.

## Figures and Tables

**Table 1:** Mean values for the 4 study groups as a result of the histopathological examination.

Histological Criteria	ABG Group	BS + PRF Group	BS Group	Control
Percentage of bone regeneration, %	85.83	71.67	54.17	3.33
Fibrous cells, %	16.67	71.67	96.67	93.33
Cartilage cells, %	18.75	17.50	3.33	6.67
Bone cells, %	64.58	10.83	0	0
Angiogenesis, vessels/mm^2^	2.09	6.69	14.58	0
Giant cell reaction, cells/mm^2^	0.7975	2.6575	3.3267	0
Chronic inflammatory infiltrate, cells/mm^2^	0.92	3.92	11.25	0
Cell remodeling, cells/mm^2^	17.00	19.33	8.83	1.25

**Table 2:** Results of the Mann–Whitney U test on quantitative callus formation.

Study Groups	Test Value	Z Statistic	*p*-Value
ABG—CONTROL	78.00	−4.269	0.000
BS—CONTROL	78.00	−4.256	0.000
BS + PRF—CONTROL	86.00	−3.824	0.000
ABG—BS	89.00	−3.563	0.000
BS + PRF—BS	109.50	−2.373	0.018
ABG—BS + PRF	123.00	−1.600	0.110

**Table 3:** Mann–Whitney U test applied to the fibrous cell count in the callus.

Study Groups	Test Value	Z Statistic	*p*-Value
ABG—BS	78.00	−4.327	0.000
ABG—control	79.00	−4.270	0.000
ABG—BS + PRF	85.00	−3.819	0.000
BS + PRF—BS	94.00	−3.415	0.001
BS + PRF—control	101.00	−2.999	0.003
BS—control	146.00	−0.304	0.843

**Table 4:** Mann–Whitney U test applied to the cartilage cell count in the callus.

Study Groups	Test Value	Z Statistic	*p*-Value
ABG—BS	96.50	−3.248	0.001
BS + PRF—BS	99.50	−3.103	0.002
ABG—control	110.50	−2.384	0.020
BS + PRF—control	114.50	−2.185	0.029
BS—control	146.00	−0.304	0.761
ABG—BS + PRF	145.00	−0.299	0.799

**Table 5:** Mann–Whitney U test applied to the bone cell count in the callus.

Study Groups	Test Value	Z Statistic	*p*-Value
ABG—BS	78.00	−4.446	0.000
ABG—control	78.00	−4.446	0.000
ABG—BS + PRF	84.50	−3.825	0.000
BS + PRF—BS	96.00	−3.616	0.001
BS + PRF—control	96.00	−3.616	0.001
BS—control	150.00	-	1.000

**Table 6:** Mann–Whitney U test applied to the blood vessel count per mm^2^.

Study Groups	Test Value	Z Statistic	*p*-Value
ABG—BS	78.00	−4.157	0.000
ABG—BS+PRF	95.50	−3.147	0.002
BS + PRF—BS	100.00	−2.887	0.004

**Table 7:** Mann–Whitney U test applied to giant cell reaction values.

Study Groups	Test Value	Z Statistic	*p*-Value
ABG—BS	88.00	−3.587	0.000
ABG—BS + PRF	101.50	−2.805	0.005
BS + PRF—BS	129.50	−1.186	0.236

**Table 8:** Mann–Whitney U test applied to the chronic inflammatory infiltrate (lymphocyte count).

Study Groups	Test Value	Z Statistic	*p*-Value
ABG—BS	78.00	−4.282	0.000
BS + PRF—BS	88.00	−3.603	0.000
ABG—BS + PRF	98.50	−3.078	0.002

**Table 9:** Mann–Whitney U test applied to the bone remodelling process.

Study Groups	Test Value	Z Statistic	*p*-Value
ABG—control	78.00	−4.215	0.000
BS+PRF—control	78.00	−4.210	0.000
BS—control	81.00	−4.041	0.000
BS + PRF—BS	86.50	−3.674	0.000
ABG—BS	88.00	−3.591	0.000
ABG—BS + PRF	127.00	−1.333	0.198

**Table 10:** Mann–Whitney U test applied to total callus volume values.

Study Groups	Test Value	Z Statistic	*p*-Value
BS—CONTROL	78.00	−4.257	0.000
ABG—CONTROL	78.00	−4.242	0.000
BS + PRF—CONTROL	78.00	−4.220	0.000
BS + PRF—BS	87.00	−3.702	0.000
ABG—BS	88.00	−3.660	0.000
ABG—BS + PRF	139.50	−0.619	0.536

## Data Availability

The raw data supporting the conclusions of this article will be made available by the authors on request.
